# A machine learning-based prognostic model for *de novo* metastatic HR-positive breast cancer: SEER cohort with external validation

**DOI:** 10.3389/fonc.2026.1875284

**Published:** 2026-07-17

**Authors:** Sihang Lin, Wanwan Wang, Lixia Liu, Jiayu Guan, Chuanrong Cen, Huawei Yang

**Affiliations:** 1 Department of Breast Surgery, Key Laboratory of Breast Cancer Diagnosis and Treatment Research of Guangxi Department of Education, Guangxi Medical University Cancer Hospital, Nanning, China; 2 The Second Clinical Medical College of Jinan University, Shenzhen, China

**Keywords:** hormone receptor-positive, machine learning, metastatic breast cancer, postoperative radiotherapy, prognostic prediction model, SEER database

## Abstract

**Objective:**

To investigate the prognostic value of postoperative radiotherapy (RT) for overall survival (OS) in patients with *de novo* metastatic hormone receptor (HR)-positive breast cancer, and to develop and externally validate a machine learning-based prognostic prediction model to support prognostic risk stratification in patients with or without postoperative radiotherapy.

**Methods:**

2,266 patients with *de novo* metastatic HR-positive breast cancer from the Surveillance, Epidemiology, and End Results (SEER) database (2010–2015) were retrospectively enrolled as the training cohort, and 79 patients from Guangxi Medical University Cancer Hospital (2015–2020) served as the independent external validation cohort. OS was compared between the RT and non-RT groups using Kaplan–Meier analysis and the log-rank test. Multivariate Cox regression was performed to identify independent prognostic factors. Four machine learning models—K-nearest neighbors (KNN), logistic regression (LR), random forest (RF), and extreme gradient boosting (XGBoost)—were constructed to predict 3−year OS and evaluated using the area under the receiver operating characteristic curve (AUC), average precision (AP), calibration curves, and decision curve analysis (DCA). Postoperative RT status was included as one of the input features in all models. The predicted outcome reflects 3-year prognostic risk (i.e., probability of death within 3 years) rather than the causal RT benefit.

**Results:**

In the training cohort, the RT group demonstrated significantly superior OS compared with the non-RT group (HR = 0.52, P < 0.001). Multivariate analysis confirmed RT as an independent protective factor for OS (HR = 0.657, P < 0.001), a finding further validated in the external validation cohort (HR = 0.171, P = 0.015). Among the four models, the LR model achieved the best performance, with an AUC of 0.721, an AP of 0.429, and a calibration slope of 0.97. DCA demonstrated favorable net clinical benefit for the LR model.

**Conclusion:**

Postoperative RT is an independent protective factor for OS in patients with *de novo* metastatic HR-positive breast cancer. The LR-based prognostic model provides reliable prognostic risk estimates and may serve as a supportive reference for clinical discussions. This model predicts observed survival risk, not the causal benefit of RT, and should not be used as a standalone tool for treatment decisions.

## 1 Introduction

Breast cancer is the most prevalent malignancy among women worldwide (1), with hormone receptor (HR)-positive tumors representing the most common molecular subtype, accounting for over 70% of all cases (2). Patients presenting with *de novo* metastatic HR-positive breast cancer have substantially worse prognosis than those with early-stage disease. Although systemic therapy constitutes the cornerstone of treatment for this population, the survival benefit of locoregional radiotherapy to the primary tumor remains highly controversial. Multiple prospective randomized controlled trials (RCTs) have demonstrated that, in the context of effective systemic therapy, locoregional treatment directed at the primary tumor in metastatic breast cancer does not improve overall survival (3–6); only the MF07–01 trial observed a marginal survival benefit after extended follow-up (7). Consequently, indiscriminately administering postoperative radiotherapy to all patients with *de novo* metastatic HR-positive breast cancer appears unwarranted, and the accurate identification of patients with different prognostic trajectories under current treatment strategies represents an urgent and unmet clinical need.

Large-scale real-world data from the Surveillance, Epidemiology, and End Results (SEER) database have provided substantial support for oncologic prognostic research, and machine learning techniques have been increasingly applied in the construction of breast cancer prognostic models (8–11). However, most existing studies have not specifically focused on the metastatic HR-positive breast cancer subtype. Recently, Li et al. developed a random survival forest model using SEER data (2010–2019) for postoperative RT risk stratification in *de novo* metastatic breast cancer, reporting an HR of 0.573 after propensity score matching and notable heterogeneity of benefit across molecular subtypes (12). However, that study lacked independent external validation and did not compare multiple machine learning algorithms. Research that combines a SEER-based training cohort with an independent external validation cohort derived from a Chinese population and systematically compares multiple algorithms to construct a prognostic prediction model remains scarce. The present study extends previous work by incorporating an external Chinese cohort and systematically comparing four algorithms with a focus on model generalizability.

The present study aimed to investigate the prognostic value of postoperative radiotherapy for overall survival in patients with metastatic HR-positive breast cancer using a large-sample SEER training cohort and an independent external validation cohort from Guangxi Medical University Cancer Hospital. Four machine learning models—K-nearest neighbors (KNN), logistic regression (LR), random forest (RF), and extreme gradient boosting (XGBoost)—were constructed and validated to provide a quantitative prognostic tool for risk stratification in patients with metastatic HR−positive breast cancer under observed treatment patterns.

## 2 Materials and methods

### 2.1 Study design and ethics

This study was a retrospective cohort study that constructed a prognostic prediction model for postoperative radiotherapy in HR-positive breast cancer based on a public database and single-center clinical data, with independent external validation. The study was conducted in accordance with the Declaration of Helsinki and approved by the Ethics Committee of Guangxi Medical University Cancer Hospital (Approval No. KY2025035). Written informed consent was obtained from all patients included in the external validation cohort. The SEER database is a publicly available, de-identified dataset; therefore, no additional ethical approval was required for the training cohort.

### 2.2 Data sources and study population

#### 2.2.1 Training cohort

Data of patients diagnosed with primary HR-positive (ER-positive and/or PR-positive) breast cancer between January 2010 and December 2015 were extracted from the Surveillance, Epidemiology, and End Results (SEER) database of the National Cancer Institute. The inclusion criteria were as follows: (1) female patients aged ≥ 18 years; (2) pathologically confirmed invasive breast cancer; (3) HR-positive disease; (4) presence of distant metastasis at initial diagnosis (M1 stage, according to the AJCC 7th edition) (13); (5) having undergone surgical treatment (breast-conserving surgery or total mastectomy); and (6) complete clinicopathological and follow-up data. The exclusion criteria were as follows: (1) male breast cancer; (2) history of other primary malignancies; and (3) missing data on key variables (e.g., T/N stage, radiotherapy information, survival outcomes). A total of 2,266 patients were ultimately included as the training cohort.

#### 2.2.2 External validation cohort

Patients with primary HR-positive breast cancer treated at Guangxi Medical University Cancer Hospital between January 2015 and December 2020 were consecutively enrolled. The inclusion and exclusion criteria were identical to those applied to the training cohort. A total of 79 patients were ultimately included as the external validation cohort.

### 2.3 Variable definitions and study endpoints

Exposure variable: Postoperative radiotherapy (RT), defined as locoregional radiotherapy administered after breast cancer surgery, was categorized as “Yes” or “No”.

Outcome variable: Overall survival (OS) was defined as the time from the date of pathological diagnosis to death from any cause. Patients who were alive or lost to follow-up were censored at the date of last contact.

Covariates: Based on a review of the literature and clinical relevance, the following variables were collected for analysis and modeling:

Demographic characteristics: Age (categorized into three groups: ≤ 50 years, 51–70 years, and > 70 years);

Tumor pathological characteristics: Histological type (invasive ductal carcinoma, invasive lobular carcinoma, other), histological grade (G1, G2, G3, G4), T stage (T1, T2, T3, T4, according to the AJCC 7th edition), N stage (N0, N1, N2, N3);

Treatment information: Chemotherapy (Yes/No);

Biomarkers: HER2 status (Negative/Positive, as determined by immunohistochemistry and/or FISH);

Distant metastasis status: Bone metastasis, brain metastasis, liver metastasis, and lung metastasis (each categorized as Yes/No, based on imaging records at the time of diagnosis).

### 2.4 Statistical analysis

All statistical analyses and graphical visualizations were performed using R software (version 4.2.1) (14). Categorical variables were presented as counts (percentages), and between-group comparisons were conducted using the chi-square test or Fisher’s exact test when theoretical frequencies were < 5. Survival curves were generated using the Kaplan–Meier method, and differences in OS between the RT and non-RT groups were compared using the log-rank test. Survival analyses were performed using the survival (15)and survminer (16) packages in R. Univariate and multivariate Cox proportional hazards regression models were employed to identify independent prognostic factors for OS. Covariates adjusted in the multivariate model included age, race, marital status, histological grade, T stage, N stage, chemotherapy, and distant metastases (bone, brain, liver, and lung). Hazard ratios (HR) and their corresponding 95% confidence intervals (CI) were calculated and presented in forest plots. All tests were two-sided, and a P value < 0.05 was considered statistically significant. The proportional hazards (PH) assumption was satisfied for the multivariate Cox regression model. No formal interaction analyses between RT and other covariates were performed. Given the retrospective nature of the data and the presence of unmeasured confounders (e.g., performance status, metastatic burden, systemic therapy details), such analyses would remain susceptible to residual bias and could produce misleading causal interpretations. We therefore adopted a prognostic modeling framework. For the machine learning prediction models, the binary outcome was defined as death within 3 years after pathological diagnosis. Patients who died within 3 years were labeled as “dead”; those who survived beyond 3 years or were censored before 3 years were labeled as “alive.” All 2,266 patients in the SEER training cohort had complete follow-up or death within the study period, ensuring unambiguous outcome labeling. For the external validation cohort, the same 3−year OS definition was applied, and all 79 patients had complete 3−year follow−up or an event within 3 years.

### 2.5 Machine learning model construction and evaluation

Using the 2,266 patients from the SEER training cohort as the training sample, all clinicopathological variables listed in Section 2.3, including postoperative RT status (Yes/No), were used as input features. The binary outcome was 3−year OS status (dead/alive), as defined in Section 2.4. It is important to note that this outcome reflects the patient’s prognostic risk (i.e., predicted probability of death within 3 years) rather than the causal benefit of RT itself. Four supervised machine learning models were constructed: K−nearest neighbors (KNN), logistic regression (LR), random forest (RF), and extreme gradient boosting (XGBoost).

The SEER training cohort was randomly split into a training set (70%, n = 1,586) and an internal validation set (30%, n = 680), stratified by RT status and 3−year OS to maintain outcome proportions. The training set was used for 10−fold cross−validation and hyperparameter tuning; final model performance was subsequently evaluated on the held−out internal validation set and, ultimately, on the independent external validation cohort (N = 79). For all metrics reported below, results are presented for the external validation cohort as the primary evaluation.

Model construction and internal training were performed using the caret package (17)in R, with RF implemented based on Breiman’s random forest algorithm (18), XGBoost via the xgboost package (19), and KNN based on the nearest neighbor classification method (20). Categorical variables were processed using one−hot encoding. Patients with missing data on key variables had been excluded during cohort selection (see Section 2.2); therefore, no imputation was required in the modeling dataset. No resampling or class-weighting techniques were applied to address class imbalance; models were trained on the original class distribution to reflect real-world prevalence. The event rate (death within 3 years) in the training cohort was approximately 22.7% (514/2266).

The following hyperparameters were tuned: for RF, mtry (number of variables randomly sampled at each split, range: 1 to number of features); for XGBoost, nrounds (50–500), max_depth (3–10), eta (0.01–0.3), and gamma (0–5); for KNN, k (1–30). The optimal hyperparameters were selected based on the highest AUC in 10-fold cross-validation. For LR, the model was fitted with L2 regularization (ridge penalty) via the glmnet engine; the regularization parameter λ was tuned over a default grid. The final selected hyperparameters were as follows: RF (mtry = 4, n_estimators = 200, max_depth = 15), XGBoost (nrounds = 200, max_depth = 6, eta = 0.05, gamma = 0), KNN (k = 15), LR (λ = 10).

#### 2.5.1 Discrimination assessment

Receiver operating characteristic (ROC) curves were plotted, and the area under the curve (AUC) was calculated. Precision–recall (PR) curves were generated, and average precision (AP) was computed. The 95% confidence intervals (CI) for AUC and AP were estimated using DeLong’s method for ROC curves and bootstrap resampling (1,000 replicates) for PR curves. Additionally, metrics including F1 score, sensitivity, and specificity were calculated, and a confusion matrix was constructed at a classification threshold of 0.5. Corresponding 95% CIs for these metrics were obtained via bootstrap.

#### 2.5.2 Calibration assessment

The predicted probabilities of each model were calibrated using Platt scaling. Calibration curves were plotted, and calibration slopes were computed. A slope closer to 1 indicates better calibration. Calibration-in-the-large and Brier scores were also reported to assess overall accuracy and calibration performance.

#### 2.5.3 Clinical utility assessment:

Decision curve analysis (DCA) was performed to calculate the net benefit of each model across threshold probabilities ranging from 0.1 to 0.9. To align with clinical relevance, particular attention was given to the range of 0.2–0.5, where decisions regarding postoperative RT are typically most ambiguous. Model performance was compared against the two extreme strategies of “treat all” and “treat none”.

## 3 Results

### 3.1 Baseline clinicopathological characteristics of patients

The SEER training cohort comprised 2,266 patients with HR-positive breast cancer, including 1,285 (56.7%) in the postoperative radiotherapy (RT) group and 981 (43.3%) in the non-RT group. Statistically significant differences were observed between the two groups in the distribution of age, histological type, N stage, chemotherapy, HER2 status, brain metastasis, liver metastasis, and lung metastasis (all P < 0.05); no significant differences were found in histological grade, T stage, or bone metastasis (**Table 1**). The proportion of patients aged ≤ 50 years (37.5% vs. 22.6%), those who received chemotherapy (86.1% vs. 72.7%), and those with HER2-positive disease (25.3% vs. 17.4%) were all higher in the RT group than in the non-RT group.

**Table 1:** Baseline clinicopathological characteristics stratified by postoperative radiotherapy in HR−positive metastatic breast cancer patients from the SEER training cohort (N = 2266).

Variable	Category	Total	No_RT	RT	P_value
Age (years)	>70	594 (26.2%)	337 (34.4%)	257 (20%)	<0.001
Age (years)	≤50	704 (31.1%)	222 (22.6%)	482 (37.5%)	
Age (years)	51-70	968 (42.7%)	422 (43%)	546 (42.5%)	
Histopathology	Ductal	1804 (79.6%)	755 (77%)	1049 (81.6%)	<0.001
Histopathology	Lobular	261 (11.5%)	147 (15%)	114 (8.9%)	
Histopathology	Other	201 (8.9%)	79 (8.1%)	122 (9.5%)	
Histologic Grade	G1	141 (6.2%)	64 (6.5%)	77 (6%)	0.584
Histologic Grade	G2	958 (42.3%)	424 (43.2%)	534 (41.6%)	
Histologic Grade	G3	964 (42.5%)	413 (42.1%)	551 (42.9%)	
Histologic Grade	G4	203 (9%)	80 (8.2%)	123 (9.6%)	
T Stage	T1	343 (15.1%)	164 (16.7%)	179 (13.9%)	0.178
T Stage	T2	843 (37.2%)	354 (36.1%)	489 (38.1%)	
T Stage	T3	507 (22.4%)	227 (23.1%)	280 (21.8%)	
T Stage	T4	573 (25.3%)	236 (24.1%)	337 (26.2%)	
N Stage	N0	432 (19.1%)	220 (22.4%)	212 (16.5%)	0.003
N Stage	N1	950 (41.9%)	405 (41.3%)	545 (42.4%)	
N Stage	N2	425 (18.8%)	176 (17.9%)	249 (19.4%)	
N Stage	N3	459 (20.3%)	180 (18.3%)	279 (21.7%)	
Chemotherapy	No	447 (19.7%)	268 (27.3%)	179 (13.9%)	<0.001
Chemotherapy	Yes	1819 (80.3%)	713 (72.7%)	1106 (86.1%)	
HER2 Status	Negative	1770 (78.1%)	810 (82.6%)	960 (74.7%)	<0.001
HER2 Status	Positive	496 (21.9%)	171 (17.4%)	325 (25.3%)	
Bone Metastasis	No	902 (39.8%)	410 (41.8%)	492 (38.3%)	0.100
Bone Metastasis	Yes	1364 (60.2%)	571 (58.2%)	793 (61.7%)	
Brain Metastasis	No	2206 (97.4%)	970 (98.9%)	1236 (96.2%)	<0.001
Brain Metastasis	Yes	60 (2.6%)	11 (1.1%)	49 (3.8%)	
Liver Metastasis	No	1927 (85%)	795 (81%)	1132 (88.1%)	<0.001
Liver Metastasis	Yes	339 (15%)	186 (19%)	153 (11.9%)	
Lung Metastasis	No	1886 (83.2%)	768 (78.3%)	1118 (87%)	<0.001
Lung Metastasis	Yes	380 (16.8%)	213(21.7%)	167(13.0%)	

Categorical variables are presented as number (percentage). Between−group comparisons were performed using the chi−square test. A P value < 0.05 was considered statistically significant.

RT, postoperative radiotherapy; HR, hormone receptor; SEER, Surveillance, Epidemiology, and End Results.

The external validation cohort comprised 79 patients, including 33 (41.8%) in the RT group and 46 (58.2%) in the non-RT group. A statistically significant difference between the two groups was observed only for chemotherapy (RT group 57.6% vs. non-RT group 95.7%, P < 0.001); all other baseline variables were well balanced (all P > 0.05, **Table 2**).

**Table 2:** Baseline clinicopathological characteristics stratified by postoperative radiotherapy in HR−positive metastatic breast cancer patients from the hospital external validation cohort (N = 79).

Variable	Category	Total	No_RT	RT	P_value
Age (years)	>70	1 (1.3%)	1 (2.2%)	0 (0%)	0.892
Age (years)	≤50	47 (59.5%)	28 (60.9%)	19 (57.6%)	
Age (years)	51-70	31 (39.2%)	17 (37%)	14 (42.4%)	
Histopathology	Ductal	73 (92.4%)	44 (95.7%)	29 (87.9%)	0.342
Histopathology	Lobular	4 (5.1%)	2 (4.3%)	2 (6.1%)	
Histopathology	Other	2 (2.5%)	0 (0%)	2 (6.1%)	
Histologic Grade	G1	10 (12.7%)	6 (13%)	4 (12.1%)	0.735
Histologic Grade	G2	39 (49.4%)	21 (45.7%)	18 (54.5%)	
Histologic Grade	G3	30 (38%)	19 (41.3%)	11 (33.3%)	
T Stage	T1	2 (2.5%)	1 (2.2%)	1 (3%)	0.952
T Stage	T2	24 (30.4%)	13 (28.3%)	11 (33.3%)	
T Stage	T3	12 (15.2%)	7 (15.2%)	5 (15.2%)	
T Stage	T4	41 (51.9%)	25 (54.3%)	16 (48.5%)	
N Stage	N0	32 (40.5%)	19 (41.3%)	13 (39.4%)	0.441
N Stage	N1	17 (21.5%)	12 (26.1%)	5 (15.2%)	
N Stage	N2	20 (25.3%)	10 (21.7%)	10 (30.3%)	
N Stage	N3	10 (12.7%)	5 (10.9%)	5 (15.1%)	
Chemotherapy	No	16 (20.3%)	2 (4.3%)	14 (42.4%)	<0.001
Chemotherapy	Yes	63 (79.7%)	44 (95.7%)	19 (57.6%)	
HER2 Status	Negative	42 (53.2%)	28 (60.9%)	14 (42.4%)	0.164
HER2 Status	Positive	37 (46.8%)	18 (39.1%)	19 (57.6%)	
Bone Metastasis	No	21 (26.6%)	11 (23.9%)	10 (30.3%)	0.707
Bone Metastasis	Yes	58 (73.4%)	35 (76.1%)	23 (69.7%)	
Brain Metastasis	No	78 (98.7%)	46 (100%)	32 (97%)	0.418
Brain Metastasis	Yes	1 (1.3%)	0 (0%)	1 (3%)	
Liver Metastasis	No	57 (72.2%)	32 (69.6%)	25 (75.8%)	0.726
Liver Metastasis	Yes	22 (27.8%)	14 (30.4%)	8 (24.2%)	
Lung Metastasis	No	62 (78.5%)	34 (73.9%)	28 (84.8%)	0.374
Lung Metastasis	Yes	17 (21.5%)	12 (26.1%)	5 (15.2%)	

Categorical variables are presented as number (percentage). The chi−square test was used for between−group comparisons, and Fisher’s exact test was applied for small sample sizes. A P value < 0.05 was considered statistically significant.

RT, postoperative radiotherapy; HR, hormone receptor.

### 3.2 Kaplan–Meier survival analysis of postoperative radiotherapy and overall survival

**Figure 1:**
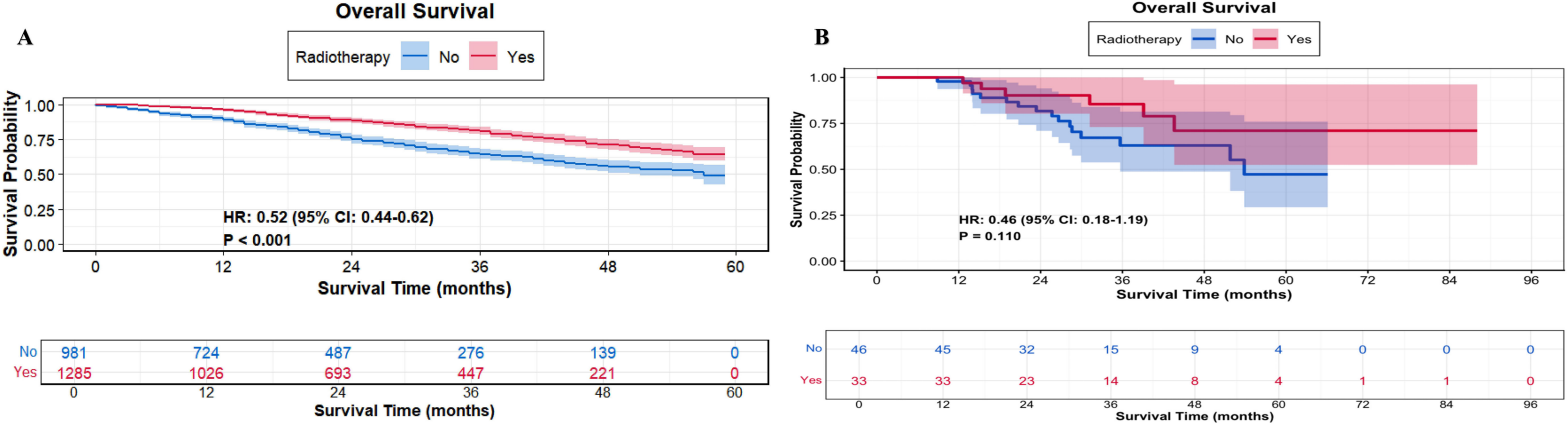
Kaplan–Meier curves of overall survival stratified by postoperative radiotherapy in patients with HR−positive metastatic breast cancer. **(A)** SEER training cohort (N = 2266); **(B)** hospital external validation cohort (N = 79). P = 0.110 by log-rank test. The number of patients at risk at each time point is shown below the x-axis. HR, hazard ratio; CI, confidence interval; RT, postoperative radiotherapy.

Kaplan–Meier analysis in the training cohort demonstrated that OS in the RT group was significantly superior to that in the non-RT group (HR = 0.52, 95% CI: 0.44–0.62, P < 0.001, **Figure 1A**). Although univariate analysis in the external validation cohort did not reach statistical significance, a trend toward better observed survival was noted for the RT group (HR = 0.46, P = 0.110, **Figure 1B**). After multivariate adjustment, postoperative radiotherapy was associated with improved overall survival in the external validation cohort.

### 3.3 Identification of independent prognostic factors by multivariate Cox regression

**Figure 2:**
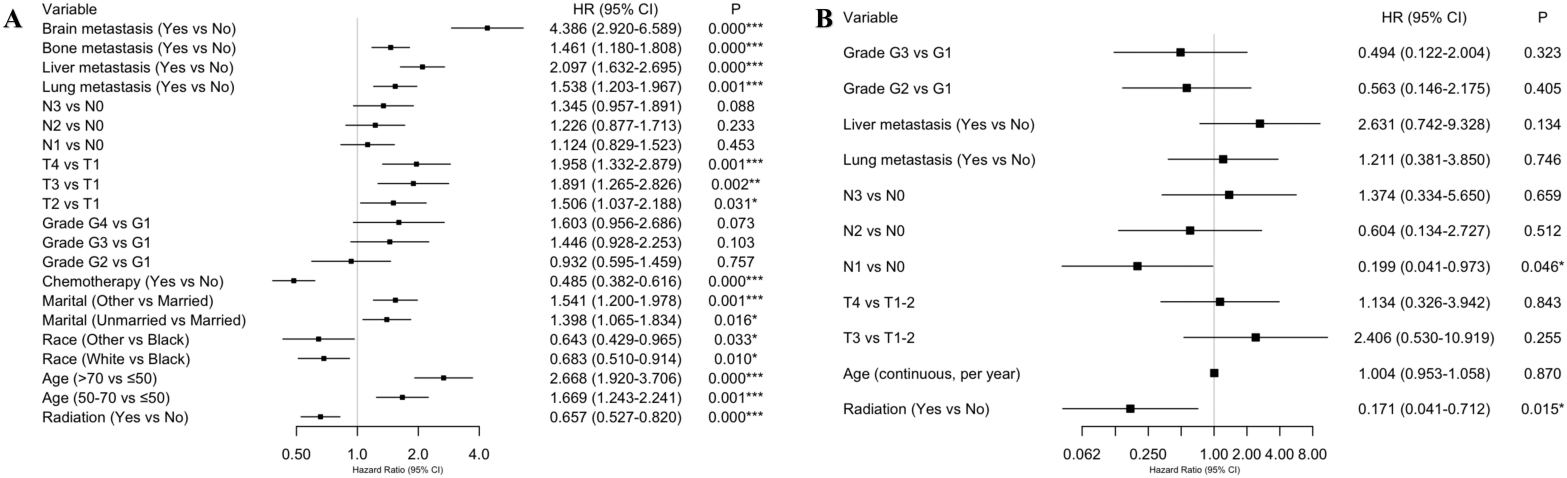
Multivariate Cox regression forest plots for overall survival in patients with HR−positive metastatic breast cancer. **(A)** SEER training cohort (adjusted for age, race, marital status, chemotherapy, grade, T stage, N stage, and distant metastases); **(B)** hospital external validation cohort (adjusted for age, T stage, N stage, grade, and distant metastases). Horizontal lines represent 95% confidence intervals; the vertical dashed line indicates HR = 1. *P < 0.05, ***P < 0.001. HR, hazard ratio; CI, confidence interval; RT, postoperative radiotherapy.

Multivariate Cox regression forest plots are presented in **Figure 2**. In the SEER training cohort, postoperative RT was identified as an independent protective factor for OS (HR = 0.657, 95% CI: 0.527–0.820, P < 0.001). Age > 70 years (vs. ≤ 50 years), T3/T4 stage (vs. T1), and the presence of bone, brain, liver, and lung metastases were all independent risk factors for OS, whereas chemotherapy was an independent protective factor (all P < 0.05, **Figure 2A**).

In the external validation cohort, after adjusting for age, T stage, N stage, histological grade, and distant metastases, postoperative radiotherapy was associated with improved overall survival, consistent with findings from the training cohort (HR = 0.171, 95% CI: 0.041–0.712, P = 0.015). Additionally, N1 stage was associated with improved survival (HR = 0.199, P = 0.046, **Figure 2B**).

### 3.4 Performance evaluation of machine learning models

#### 3.4.1 Discrimination assessment

**Figure 3:**
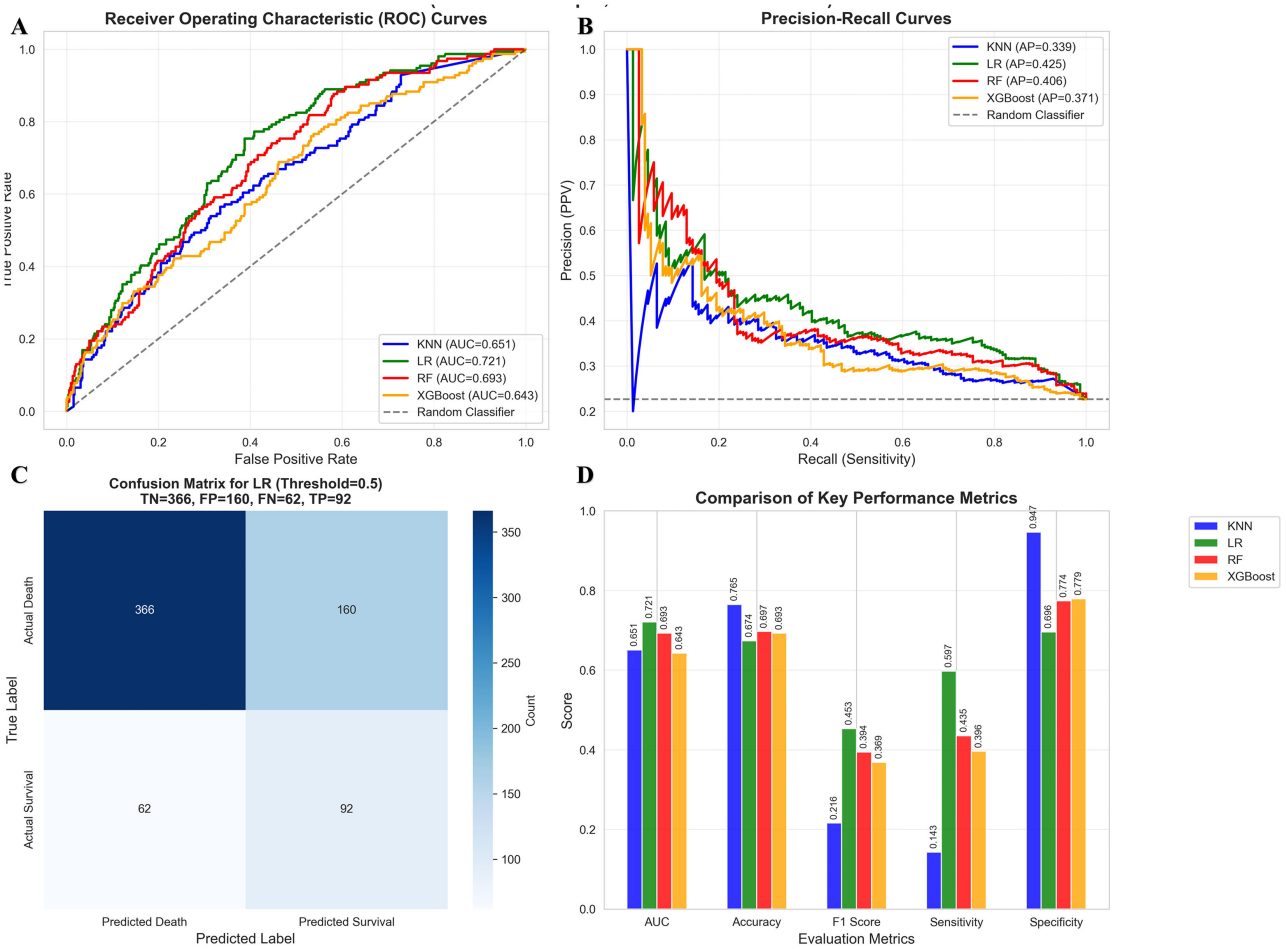
Performance evaluation of four machine learning models for predicting overall survival. **(A)** Receiver operating characteristic (ROC) curves; **(B)** precision–recall (PR) curves; **(C)** confusion matrix of the logistic regression (LR) model at a classification threshold of 0.5; **(D)** comparison of key evaluation metrics. AUC, area under the ROC curve; AP, average precision; KNN, k−nearest neighbor; LR, logistic regression; RF, random forest; XGBoost, extreme gradient boosting.

The ROC and PR curves of the four machine learning models are shown in **Figure 3**. The logistic regression (LR) model demonstrated the highest discriminative ability, with an AUC of 0.721 (95% CI: 0.678–0.764), followed by random forest (RF, AUC = 0.693, 95% CI: 0.648–0.737), K-nearest neighbors (KNN, AUC = 0.651, 95% CI: 0.604–0.695), and extreme gradient boosting (XGBoost, AUC = 0.643, 95% CI: 0.588–0.690; **Figure 3A**). In the PR curve analysis, the LR model also achieved the highest average precision (AP = 0.429, 95% CI: 0.354–0.502; **Figure 3B**). At a classification threshold of 0.5, the LR model yielded an F1 score of 0.452 (95% CI: 0.395–0.510), sensitivity of 0.596 (95% CI: 0.520–0.673), and specificity of 0.696 (95% CI: 0.660–0.733), outperforming the other models in overall discriminative performance (**Figures 3C, D**).

#### 3.4.2 Calibration assessment

**Figure 4:**
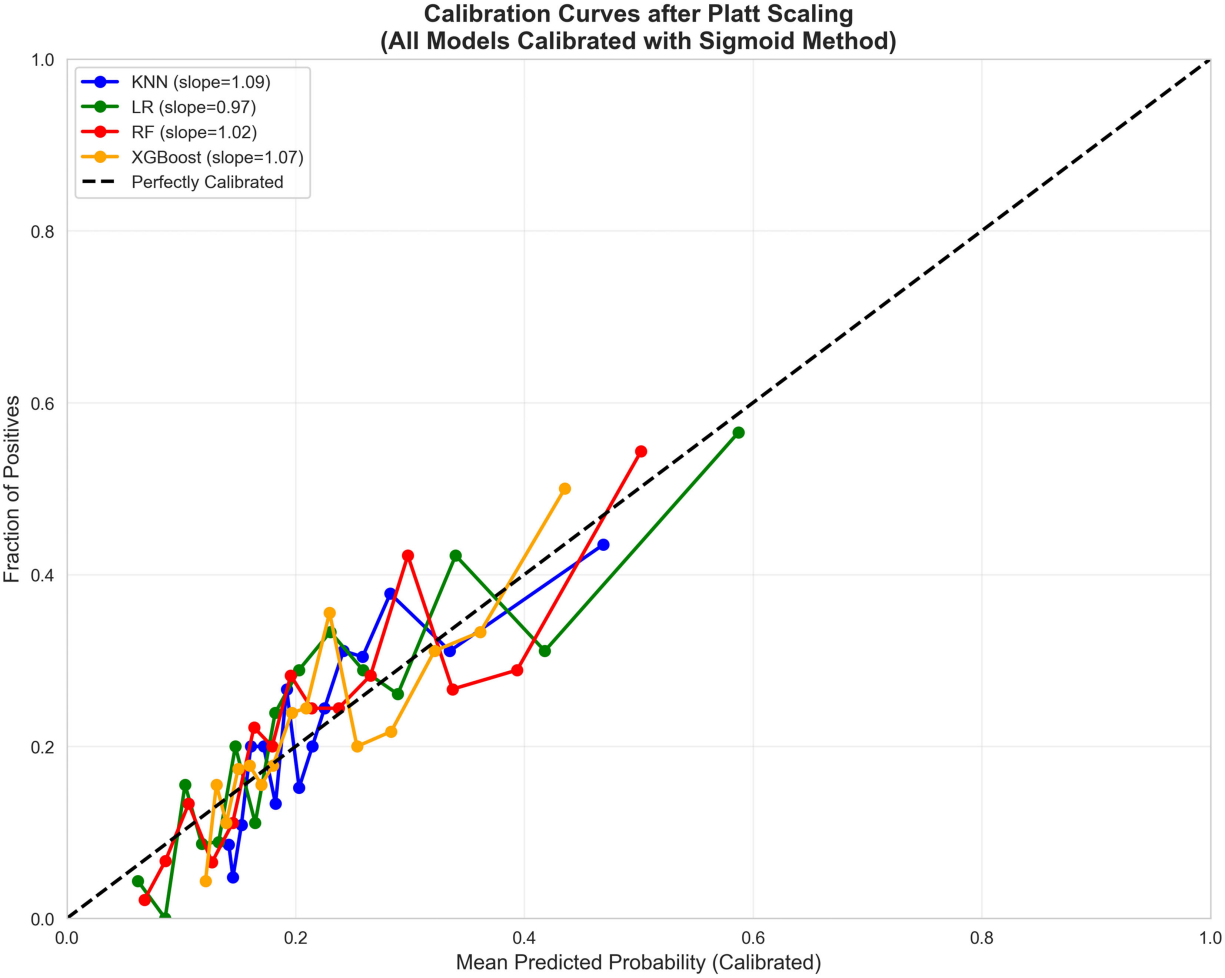
Calibration curves of four machine learning models after Platt scaling. The black dashed line indicates perfect calibration. A slope closer to 1 represents better calibration. LR, logistic regression; KNN, k−nearest neighbor; RF, random forest; XGBoost, extreme gradient boosting.

After calibration with Platt scaling, the LR model achieved a calibration slope of 0.97, which was the closest to the ideal reference line (slope = 1) among all models, indicating good calibration. The Brier score for the LR model was 0.209, and calibration-in-the-large was −2.83, confirming acceptable overall calibration. The calibration slopes of the remaining models deviated slightly from 1 (**Figure 4**).

#### 3.4.3 Clinical utility assessment

**Figure 5:**
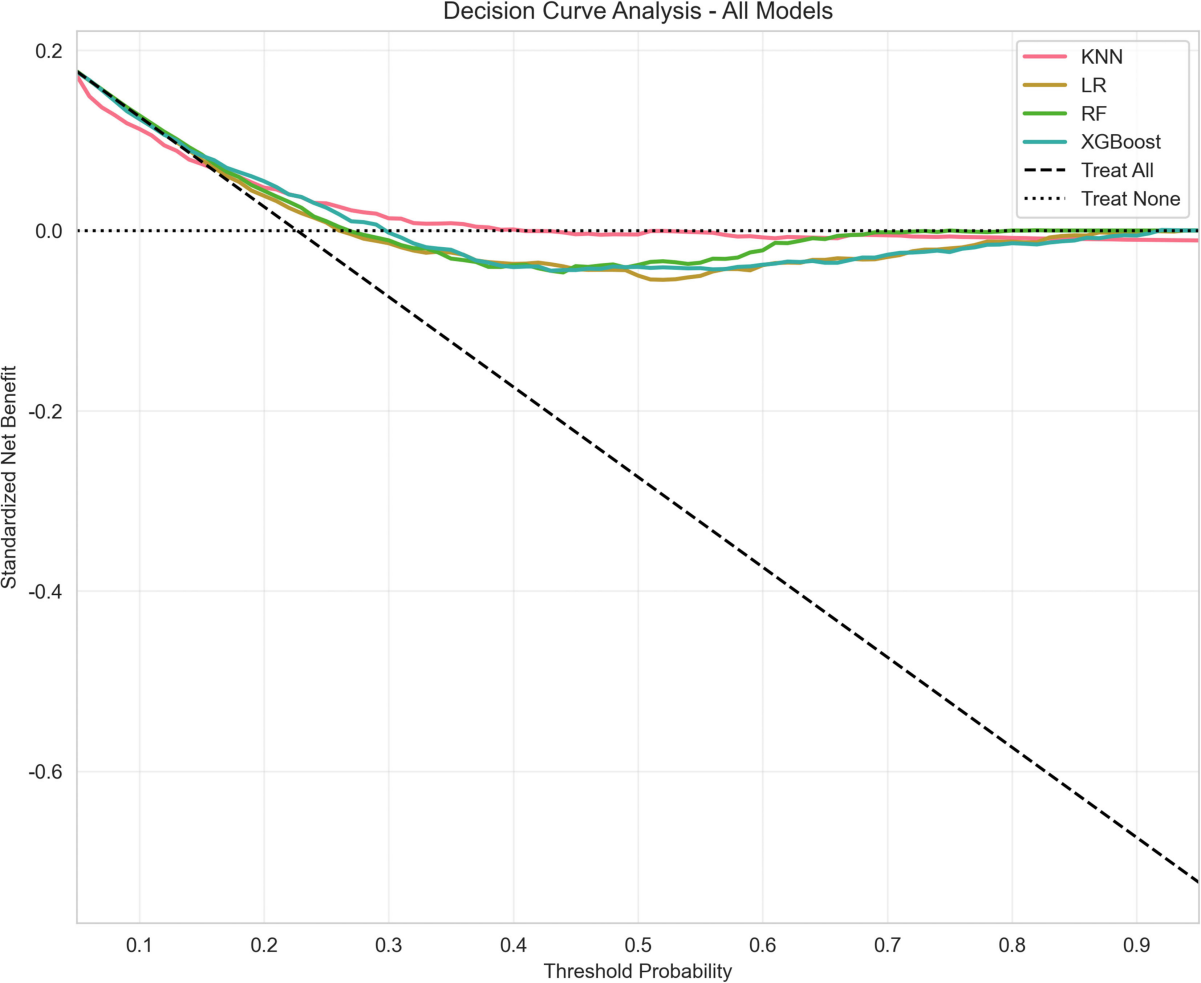
Decision curve analysis (DCA) of four machine learning models. The dashed line indicates the “Treat All” strategy; the dotted line indicates the “Treat None” strategy. KNN, k−nearest neighbor; LR, logistic regression; RF, random forest; XGBoost, extreme gradient boosting.

Decision curve analysis (DCA) demonstrated that the LR model provided a higher net clinical benefit than either the “treat all” or “treat none” strategies across a wide range of threshold probabilities. The net benefit was particularly stable in the clinically relevant range of 0.2–0.5, where decisions regarding postoperative RT are typically most ambiguous. Similar but slightly lower net benefit was observed for the other models (**Figure 5**).

## 4 Discussion

This study, leveraging a large-sample SEER training cohort combined with single-center external validation from a Chinese population, showed an independent association between postoperative radiotherapy and better overall survival in patients with metastatic HR-positive breast cancer. Furthermore, an LR prediction model with optimal comprehensive performance was constructed. Multivariate Cox regression demonstrated that postoperative RT was an independent protective factor for OS (training cohort: HR = 0.657, 95% CI: 0.527–0.820, P < 0.001), a finding that was further validated in the external validation cohort (HR = 0.171, 95% CI: 0.041–0.712, P = 0.015). Among the four machine learning models, the LR model exhibited the best discrimination, calibration, and net clinical benefit, and may serve as a supportive tool for prognostic risk stratification to inform multidisciplinary discussion regarding postoperative RT.

### 4.1 Value of postoperative radiotherapy in metastatic HR+ breast cancer and selection bias

In this study of patients with *de novo* M1 HR−positive breast cancer, we observed that those who received primary-site radiotherapy had significantly superior OS compared with those who did not (HR = 0.52), and RT remained independently associated with better OS after multivariate adjustment (HR = 0.657). This finding aligns with results from several real-world studies based on large databases such as SEER, which tend to demonstrate a favorable survival association of locoregional therapy (LRT) in M1 breast cancer.

However, this association must be interpreted with caution. The highest-level evidence from prospective randomized controlled trials (RCTs) has almost unanimously failed to confirm an OS benefit of LRT. The Tata Memorial trial was the first RCT to demonstrate that locoregional treatment of the primary tumor did not improve OS in M1 patients (3). Subsequently, the ECOG-ACRIN E2108 trial further revealed that, in patients with *de novo* M1 breast cancer who responded to initial systemic therapy, early local therapy not only failed to improve OS but might even have a detrimental effect on survival (5). The ABCSG-28 trial (4) and the JCOG1017 trial (6) similarly yielded negative results. Among the five prospective RCTs, only the MF07–01 trial observed a marginal survival benefit after extended 10-year follow-up, which was confined to specific subgroups (e.g., patients with isolated bone metastases, younger age, or HR+ disease) (7).

The striking discrepancy between observational studies and RCT results most likely stems from “confounding by indication” and “selection bias” that are difficult to measure: in real-world clinical practice, physicians are more inclined to administer primary-site radiotherapy to patients with good performance status (high PS score), low metastatic burden (e.g., bone-only or oligometastatic disease), and favorable response to systemic therapy—all of which are themselves strong indicators of a better prognosis. The SEER database notably lacks crucial prognostic variables such as performance status, the exact number of metastatic lesions, specific systemic therapy regimens, and treatment response. Consequently, the independent protective effect of radiotherapy observed in this study may be confounded by a strong patient selection effect, and its magnitude may be overestimated.

Nevertheless, in the absence of guidance from prospective biomarkers, clinicians still require tools to assist in this complex decision-making process. The LR prediction model constructed in this study can essentially be viewed as an objective quantification of the above-mentioned “selection criteria,” integrating readily available clinicopathological characteristics—such as age, T/N stage, histological grade, and metastatic sites—into a comprehensive prognostic assessment framework. This provides an objective reference for the current decision-making paradigm, which largely relies on clinical experience. However, its true predictive value—namely, whether patients identified by the model as “high-risk” show more favorable observed survival when receiving postoperative RT than when not—requires further validation in prospective studies.

The results from the external validation cohort require particularly cautious interpretation. In the univariate KM analysis, the difference in survival between RT and non-RT groups did not reach statistical significance (HR = 0.46, P = 0.110), which may be attributable to the substantial prognostic heterogeneity inherent in M1 patients and the limited number of events in a small sample (N = 79). After multivariable adjustment, the association between RT and OS became statistically significant (HR = 0.171, 95% CI: 0.041–0.712, P = 0.015); however, the wide confidence interval and small sample size indicate substantial statistical instability. Chemotherapy was significantly imbalanced between groups (57.6% in the RT group vs. 95.7% in the non-RT group, P < 0.001), and although the multivariable model adjusted for this confounder, residual confounding cannot be excluded. Given these limitations, this result should be regarded as exploratory rather than confirmatory.

It is critical to emphasize that this model is intended solely as a supportive reference to aid multidisciplinary decision-making, not as a substitute for comprehensive clinical evaluation. Given the absence of key prognostic variables—including endocrine therapy details, Ki-67, performance status, and metastatic burden—the model should be regarded as exploratory and hypothesis-generating. Overinterpretation of its predictive ability should be avoided.

### 4.2 Model performance, comparison, and clinical utility

In terms of model performance, the LR model demonstrated the best comprehensive performance (AUC = 0.721) among the four machine learning algorithms. In contrast, the more complex ensemble learning algorithms (RF, XGBoost) did not yield performance improvements but rather a slight decline. The AUC was 0.693 for RF and 0.643 for XGBoost, both lower than that of LR. This phenomenon may be related to the relatively limited feature dimensionality in this study: when input features predominantly consist of low-dimensional categorical variables, complex non-linear models cannot leverage their inherent advantage of capturing high-order interactions, and may instead introduce additional variance through overparameterization, thereby impairing their generalizability on independent external validation sets (21). Similar phenomena have been reported in clinical prediction model studies based on the SEER database. For example, a study by Gao et al., which developed a machine learning model for predicting bone metastasis in breast cancer using SEER data, also found that RF and XGBoost did not demonstrate an overwhelming advantage over LR (9).

Our findings should be viewed in the context of the recent study by Li et al., who also utilized SEER data (2010–2019) to develop a random survival forest model for postoperative RT risk stratification in 1,392 patients with dnMBC (12). After propensity score matching, they reported a significant OS benefit of postoperative RT (HR = 0.573) and substantial heterogeneity across molecular subtypes, with patients with HR+/HER2− disease deriving significant benefit while those with HER2+ disease did not. The present study extends this prior work in several respects: (1) we provide independent external validation from a Chinese cohort, whereas Li et al. relied solely on internal SEER data splitting; (2) we systematically compare multiple machine learning algorithms and find that a simpler LR model achieves better generalizability on external data than more complex ensemble methods; and (3) we explicitly frame our model as a prognostic tool rather than a treatment-benefit prediction model. Both studies converge on a common message: conventional clinicopathological variables alone are insufficient for highly accurate individualized prediction, and the integration of molecular markers and detailed treatment variables is essential for future model improvement.

The PR curve analysis in the present study further revealed that although the AP (0.429) of the LR model was superior to the other models, the absolute value remained at a moderate level, which is partly related to the relatively low incidence of OS events and the resulting class imbalance in HR-positive breast cancer. Nonetheless, after Platt scaling calibration, the calibration slope of the LR model (0.97) was close to the ideal value of 1, indicating good consistency between predicted probabilities and observed probabilities. This is critical for clinical decision-making—a model with good calibration but moderate discrimination can still provide reliable absolute risk estimates to aid treatment decisions. DCA results demonstrated that the LR model achieved a positive net benefit over the “treat all” and “treat none” strategies, with particularly stable net benefit in the clinically relevant threshold range of 0.2–0.5, where decisions regarding postoperative RT are typically most ambiguous.

It is important to emphasize that this model is intended solely as a supportive reference to aid multidisciplinary decision-making, not as a substitute for comprehensive clinical evaluation. The model predicts 3-year prognostic risk based on readily available clinicopathological features, thereby providing an objective quantitative reference when discussing postoperative RT. Based on the DCA results, threshold probabilities in the range of 0.2–0.4 may represent a clinically relevant decision range where the model provides net benefit. However, given the absence of critical prognostic variables—including endocrine therapy details, Ki-67 index, performance status, and metastatic burden—treatment decisions should not rely solely on model output.

In an indirect comparison with other studies, Zheng et al. constructed a nomogram for predicting postoperative overall survival in breast cancer using more comprehensive patient data from the SEER database (N = 394,503), achieving superior discrimination with an AUC above 0.80 (11). The performance of that model is higher than that of the LR model in the present study, primarily attributable to its larger sample size, more comprehensive variable set, and more refined subtype-specific modeling strategy. This comparison, together with the findings of Li et al. (12), suggests that the performance of our model could be further improved by expanding the sample size and incorporating endocrine therapy details and molecular markers such as Ki-67.

### 4.3 Limitations

This study has several limitations. First, as a retrospective study design, it carries inherent risks of bias. Although known confounders were adjusted for using multivariate Cox regression, the influence of unmeasured or unknown confounders (such as comorbidity index, surgical margin status, specific radiation dose and technique, and patient performance status) could not be completely ruled out; therefore, the level of evidence of this study still requires prospective validation. Second, the external validation cohort had a relatively small sample size (N = 79) and was derived from a single center (Guangxi Medical University Cancer Hospital), which may limit a thorough assessment of the model’s generalizability. Future validation is warranted in larger, multi-center prospective cohorts. Third, both the SEER database and the external validation data lacked detailed information on endocrine therapy regimens (e.g., differences between tamoxifen and aromatase inhibitors, treatment adherence, and duration) as well as important molecular pathological markers (e.g., the Ki-67 proliferation index). This represents a critical information gap in HR-positive breast cancer and may restrict further improvement in the model’s predictive accuracy. Fourth, an AUC of 0.721 indicates that the model’s discrimination is at an acceptable but not excellent level, and there remains room for improvement in identifying truly high-risk patients. Fifth, there were baseline imbalances in multiple variables between the RT and non-RT groups in the training cohort (e.g., age, HER2 status, chemotherapy proportion, and distribution of distant metastases). Although statistical adjustment was performed in the multivariate model, residual confounding could not be completely eliminated, which may affect the robustness of the conclusions to some extent. In summary, as a retrospective observational study, it is subject to selection bias, and the conclusions require validation in prospective studies. This study was conducted in accordance with the TRIPOD (Transparent Reporting of a multivariable prediction model for Individual Prognosis Or Diagnosis) statement for model development and validation. Given the above limitations, the model should be positioned as an exploratory decision-support tool that requires further validation before clinical implementation. In particular, radiotherapy-related parameters such as dose, target volume, and technique were unavailable, further limiting interpretation of treatment-related effects.

### 4.4 Future directions

Future research may be advanced in the following directions: first, incorporating detailed endocrine therapy information and molecular markers such as Ki-67 and multigene expression profiles to construct a more refined comprehensive prediction model; second, conducting multi-center, large-sample real-world external validation to assess the model’s generalizability across diverse populations; third, where feasible, employing propensity score matching (PSM) or instrumental variable analysis as sensitivity analyses to better control for confounding bias in retrospective data; and fourth, conducting prospective clinical studies to further validate the clinical utility of the model and delineate its incremental value in real-world clinical decision-making pathways.

## 5 Conclusion

In this large-sample real-world study, postoperative radiotherapy was independently associated with better overall survival in patients with HR-positive metastatic breast cancer; however, this association may be influenced by selection bias that is difficult to fully adjust for. The logistic regression-based prognostic prediction model, constructed using routine clinicopathological characteristics, provides a preliminary quantitative tool to support risk stratification when discussing postoperative RT. Its clinical utility requires further validation in prospective studies with more comprehensive clinicopathological and treatment-related variables.

## Data Availability

The raw data supporting the conclusions of this article will be made available by the authors, without undue reservation.
